# Discovery and Biological Evaluation of a Series of Pyrrolo[2,3-*b*]pyrazines as Novel FGFR Inhibitors

**DOI:** 10.3390/molecules22040583

**Published:** 2017-04-05

**Authors:** Yan Zhang, Hongchun Liu, Zhen Zhang, Ruifeng Wang, Tongchao Liu, Chaoyun Wang, Yuchi Ma, Jing Ai, Dongmei Zhao, Jingkang Shen, Bing Xiong

**Affiliations:** 1 School of Pharmaceutical Sciences, Binzhou Medical University, Yantai 264003, Shandong, China; zhangyanzy2014@126.com (Y.Z.); ytwcy@163.com (C.W.); 2 Division of Anti-Tumor Pharmacology, State Key Laboratory of Drug Research, Shanghai Institute of Materia Medica, Chinese Academy of Sciences, 555 Zuchongzhi Road, Shanghai 201203, China; hchliu@simm.ac.cn; 3 Department of Medicinal Chemistry, State Key Laboratory of Drug Research, Shanghai Institute of Materia Medica, Chinese Academy of Sciences, 555 Zuchongzhi Road, Shanghai 201203, China; 18204012193@163.com (Z.Z.); rfwang1992@126.com (R.W.); Tongchao_Liu@163.com (T.L.); jkshen@simm.ac.cn (J.S.); 4 Key Laboratory of Structure-Based Drug Design and Discovery of Ministry of Education, Shenyang Pharmaceutical University, Shenyang 110016, China; dongmeiz-67@163.com

**Keywords:** FGFR1, inhibitor, kinase inhibitor, pyrazine, hinge binder

## Abstract

Abnormality of fibroblast growth factor receptor (FGFR)-mediated signaling pathways were frequently found in various human malignancies, making FGFRs hot targets for cancer treatment. To address the consistent need for a new chemotype of FGFR inhibitors, here, we started with a hit structure identified from our internal hepatocyte growth factor receptor (also called c-Met) inhibitor project, and conducted a chemical optimization. After exploring three parts of the hit compound, we finally discovered a new series of pyrrolo[2,3-*b*]pyrazine FGFR inhibitors, which contain a novel scaffold and unique molecular shape. We believe that our findings can help others to further develop selective FGFR inhibitors.

## 1. Introduction

**Figure 1:**
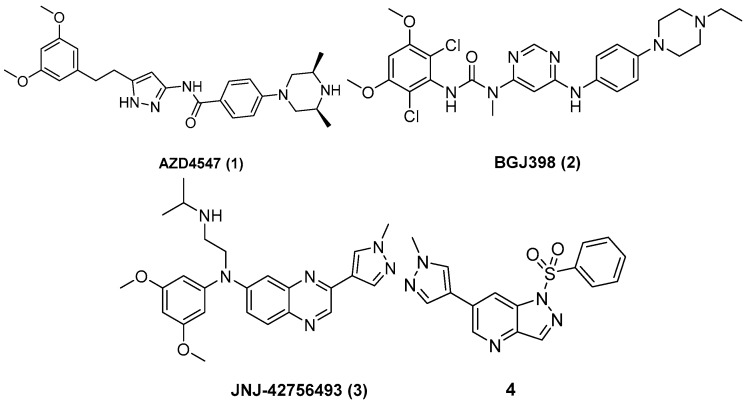
Representative selective fibroblast growth factor receptor inhibitors.

In the human genome, a total of 22 fibroblast growth factors (FGFs) have been identified, and there are four fibroblast growth factor receptors (FGFRs) that respond to and mediate the signaling from fibroblast growth factors [1,2,3,4]. Increasing evidence highlights the importance of FGFR signaling pathways in the regulation of several basic biologic processes, including tissue development, angiogenesis, and tissue regeneration [5,6]. Not surprisingly, abnormal signaling involving FGFRs has been frequently found in various human malignancies, making FGFRs hot targets in anticancer drug development [7,8,9]. Aberrant FGFR signaling could be caused by different mechanisms, including activating mutations in FGFRs, oncogenic fusion of FGFRs and over-expression of FGFRs [10]. Currently, several FGFR-targeted agents, mostly small molecules binding to the kinase domain, are being evaluated in clinical trials for cancer treatment, and the most intriguing and advanced evaluations are for FGFR-selective inhibitors, such as AZD4547 (**1**) [11], NVP-BGJ398 (**2**) [12] and JNJ-42756493 (**3**) (Figure 1) [13]. As investigated by Patani et al. [14], due to the landscape of activated mutations in FGFR kinases, the above-mentioned FGFR inhibitors (**1**, **2**, **3**) exhibited distinct effects on different mutants, which indicates that developing FGFR inhibitors with novel scaffolding are highly in demand, as they may provide unique therapeutic benefits for certain patients.

In the development of tyrosine kinase inhibitors, we previously synthesized a series of 1-sulfonylpyrazolo[4,3-*b*]pyridines as potent and selective c-Met (hepatocyte growth factor receptor) inhibitors [15]. Since c-Met and FGFR all belong to the receptor tyrosine kinase subfamily, we also tested a subset of this series of compounds in a fibroblast growth factor receptor 1 (FGFR1) enzymatic assay and serendipitously found that compound **4** has weak but definite FGFR1 activity, with an 87.8% inhibition ratio towards FGFR1 at 10 μM concentration. Starting from this compound, in the current study, we present our structure optimization and elaborated the structure–activity relationship (SAR) of this novel scaffold, in the hope that the investigation can stimulate new ideas for developing selective FGFR inhibitors as anticancer drugs.

## 2. Results and Discussion

**Figure 2:**
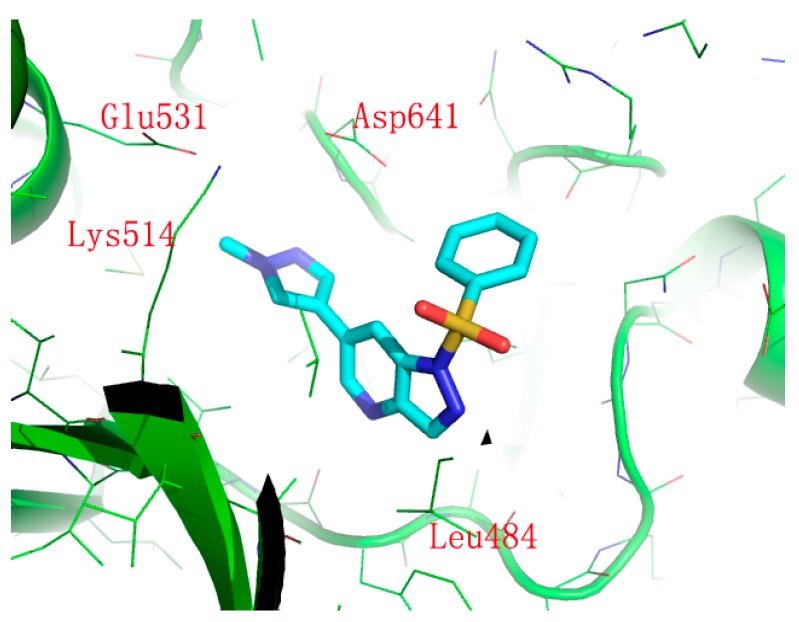
The docking study of compound **4** in FGFR1 binding site. The protein structure was extracted from the crystal structure (PDB code: 3TT0) and is shown in green, while compound **4** was depicted with a stick model and in cyan.

As reported in c-Met inhibitor development [15], the 1-sulfonylpyrazolo[4,3-*b*]pyridine acts as the hinge binder, and the benzene group of compound **4** forms a π–π interaction with the residue Tyr1230 in the c-Met ATP binding site. Since compound **4** is a novel FGFR inhibitor, both from the view of chemical ring system and from the shape of the structure, we need to probe its binding mode. Therefore, we performed a docking study to predict the binding mode of compound **4** in the FGFR1 ATP binding site prepared from a PDB (Protein Data Bank) structure (PDB code: 3TT0) [16]. As shown in Figure 2, the docking results indicated that the pyrazolo[4,3-*b*]pyridine ring still acts as the hinge binder to anchor the molecule in the ATP binding site of FGFR1. Therefore, the benzene group is located at the middle of the ATP binding site and the linking sulfamide group makes it perpendicular to the pyrazolo[4,3-*b*]pyridine. We inspected the predicted complex structure of **4** bound to FGFR1 kinase and did not find any possible residues could form the π–π interaction with the benzene group of compound **4**. Then, we designed three other compounds by modifying the connecting group of sulfamide. As shown in Table 1, three compounds showed moderate inhibitions towards FGFR1, but the activities are all inferior to the starting compound **4**. This indicated that the nearly right angle between the benzene group and pyrazolo[4,3-*b*]pyridine ring is important to the binding affinity. Together with the unique shape of compound **4**, we wanted to retain this connecting group for late optimization.

Most of the tyrosine kinase inhibitors comprise a hydrogen bonding acceptor to interact with the hinge part of the kinase domain, which is to mimic the binding pattern of the adenine group of the ATP molecule [17]. Since the hinge binder forms hydrogen bonding and Van der Waals interactions with the binding site residues, it makes a great contribution to anchoring the whole inhibitor in the kinase domain. Therefore, we modified it with the aim of improving the binding activity. We retained the important nitrogen atom as a hydrogen bond receptor to the backbone of the residue ALA564 and synthesized three compounds listed in Table 2. Changing the scaffold into the 1*H*-pyrrolo[3,2-*b*]pyridine ring (**8**) slightly decreased the binding activity, while changing into 5*H*-pyrrolo[2,3-*b*]pyrazine (**9**) dramatically increase the binding activity. Even at a concentration of 1 μM, it still showed more than 90% inhibition in the FGFR1 enzymatic assay [18,19]. We also changed the bicyclic scaffold into monocycle ring by opening the pyrrole (**10**), and the activity dropped by about 10-fold, as indicated by the inhibition ratio in Table 2.

Although we did not observe any possible residues could make direct interaction with the benzene group of compound **4** by looking at the cocrystal structure (PDB code: 3TT0), we are aware that the protein kinase domain has a complex conformational landscape, which has been extensively studied via crystal structure analyses and computer simulations [20,21,22]. Then, by analogy to developing c-Met inhibitors, we optimized the benzene group and synthesized over a dozen compounds listed in Table 3, and indeed found that this part is critical to the binding affinity. Substitution of the benzene group with saturated cyclopentane (**11**) or cyclohexane (**12**) dramatically decreased the binding activity. Generally, the electronic properties of substituents on benzene group are not essential to the binding, if comparing compounds **17** and **19** to compound **9**. These compounds are showing similar sub-micromolar enzymatic activities. By scrutiny of the structure–activity relationship, we found that the steric characteristics may be more important, and the meta position on the benzene sulfamide is favorable for the binding, which is evident from the comparison of compounds **17** and **18**, or **19** and **20**. The slightly large substitution such as acetyl group (**21**) on meta position of benzene is also tolerated, while larger propionyl group (**22**) decreased the activity. These reinforce that the steric characteristics of the inhibitors are vital to the binding interactions. From the exploration of this part, we found that the compound containing nitrobenzene group (**17**) stood out as a potent inhibitor with enzymatic activity about 85 nM. Then, this substructure pattern was retained and the methyl pyrazole group was subjected to further optimization.

To probe the SAR around the methyl pyrazole part, we synthesized analogs **23**–**25** by substituting the methyl group with different acyl groups. When R group is 1-(1*H*-pyrazol-1-yl)ethan-1-one (**23**) or 1-(1*H*-pyrazol-1-yl)propan-1-one (**24**), it showed reduced activity, giving less than a 40% inhibition ratio at the concentration of 0.1 μM (Table 4). While the compound with 1-(1*H-*pyrazol-1-yl)propan-2-one (**25**) has the similar activity to compound **17**. Modifying the methyl pyrazole to benzene groups (**26**, **27**) follows a similar trend, in that the phenylmethanol substitution (**27**) is more active than the acetophenone substitution (**26**). In general, the modification at the methyl pyrazole part did not improve the binding activity, and only **25** and **27** were found to have a similar potency to compound **17**, with IC_50_ values of 45 nM and 113 nM, respectively.

Since this series of compounds started from the project of developing c-Met inhibitors, we wanted to assess the selectivity of this series. Therefore, three potent compounds were picked to test in c-Met enzymatic assay. As shown in Table 5, three compounds demonstrated nearly no inhibition towards c-Met even at concentration of 1μM, indicating compounds of this series may be selective FGFR inhibitors.

## 3. Materials and Methods

### 3.1. Elisa Kinase Assay

The effects of compounds on the activities of indicated (FGFR1 and c-Met) kinases were determined using enzyme-linked immunosorbent assays (ELISAs) with purified recombinant proteins [19]. Briefly, 20 μg/mL poly (Glu, Tyr)_4:1_ (Sigma, St. Louis, MO, USA) was pre-coated in 96-well plates as a substrate. A 50-μL aliquot of 10 μmol/L ATP solution diluted in kinase reaction buffer (50 mmol/L HEPES (pH 7.4), 50 mmol/L MgCl_2_, 0.5 mmol/L MnCl_2_, 0.2 mmol/L Na_3_VO_4_, and 1 mmol/L DTT) was added to each well; 1 μL of various concentrations of compounds diluted in 1% DMSO (*v*/*v*) (Sigma) were then added to each reaction well. DMSO (1%, *v*/*v*) was used as the negative control. The kinase reaction was initiated by the addition of purified tyrosine kinase proteins FGFR1 (Millipore, Darmstadt, Germany) or c-Met (Millipore) diluted in 49 μL of kinase reaction buffer. After incubation for 60 min at 37 °C, the plate was washed three times with phosphate-buffered saline (PBS) containing 0.1% Tween 20 (T-PBS). Anti-phosphotyrosine (PY99) antibody (Sanra Cruz, Dallas, USA) (100 μL; 1:500, diluted in 5 mg/mL bovine serum albumin (BSA) T-PBS) was then added. After a 30-min incubation at 37 °C, the plate was washed three times, and 100 μL horseradish peroxidase-conjugated goat anti-mouse IgG (Calbiochem, Shanghai, China) (1:2000, diluted in 5 mg/mL BSA T-PBS) was added. The plate was then incubated at 37°C for 30 min and washed 3 times. A 100-μL aliquot of a solution containing 0.03% H_2_O_2_ and 2 mg/mL *o*-phenylenediamine in 0.1 mol/L citrate buffer (pH 5.5) was added. The reaction was terminated by the addition of 50 μL of 2 mol/L H_2_SO_4_ as the color changed, and the plate was analyzed using a multi-well spectrophotometer (SpectraMAX 190, Molecular Devices, Palo Alto, CA, USA) at 490 nm. The inhibition rate (%) was calculated using the following equation:

IR = [1 − (A_490_/A_490_control)] × 100%. (1)

where IR, A_490_ and A_490_control are the inhibition rate, the absorbance value of the tested compound at 490 nm and the absorbance value of the negative control compound at 490 nm, respectively. The IC_50_ values were calculated from the inhibition curves in two separate experiments.

### 3.2. Docking Study

The BGJ398-bound FGFR1 complex structure (PDB code: 3TT0) was downloaded from the PDB database, and prepared with protein preparation module in Schrödinger software package (Schrödinger, New York City, NY, USA). Then the Glide software (Schrödinger) was used to build the grid file within FGFR1 ATP binding site. Compound **4** was minimized with an OPLS2015 force field, and then it was docked into the FGFR1 ATP site with default XP precision parameters implemented in Glide software. Finally, the best predicted binding conformation of compound **4** was illustrated with the Pymol program (Schrödinger).

### 3.3. Chemistry

**Scheme 1:**
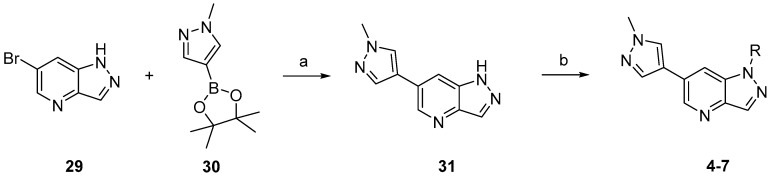
The synthesis route of compounds **4–7**. Reagents and conditions: (**a**) Pd(dppf)Cl_2_, K_2_CO_3_, Dioxane:H_2_O (*v*/*v* = 4:1), 80 °C, 2 h, 86% yield; (**b**) NaH, Dimethylformamide (DMF), R-SO_2_Cl, room temperature (r.t.), 1–4 h, 76–83% yield.

Compounds **4**–**7** were synthesized according to the procedures outlined in Scheme 1. Suzuki coupling of commercially available **29** with 1-methylpyrazole-4-boronic acid pinacol ester (**30**) provided **31**. Compounds **4**–**7** were prepared by deprotonation of compound **31** followed by addition of the corresponding electrophile reagents.

**Scheme 2:**
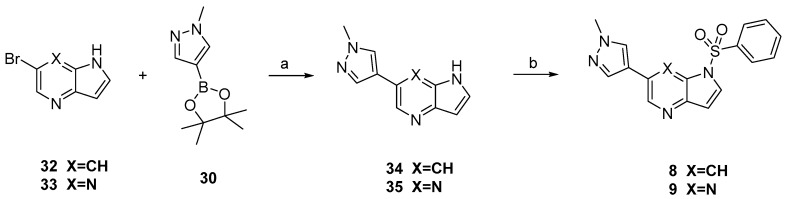
The synthesis route of compounds **8** and **9**. Reagents and conditions: (**a**) Pd(dppf)Cl_2_, K_2_CO_3_, Dioxane:H_2_O (*v*/*v* = 4:1), 80 °C, 3 h, 89% yield (**34**), 82% yield (**35**); (**b**) NaH, DMF, benzenesulfonyl chloride, r.t., 2 h, 82% yield (**8**), 79% yield (**9**).

Compounds **8** and **9** were prepared according to the procedure in Scheme 2. Treatment of compounds **32** and **33** with **30** via Suzuki coupling afforded compounds **34** and **35** which were sulfonylated to afford **8** and **9**, respectively.

**Scheme 3:**
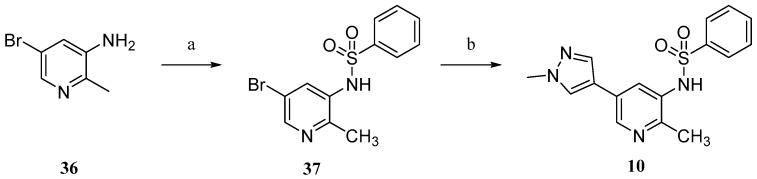
The synthesis route of compound **10**. Reagents and conditions: (**a**) benzenesulfonyl chloride, NaH, DMF, r.t., 85% yield; (**b**) 1-Methylpyrazole-4-boronic acid pinacol ester, Pd(dppf)Cl_2_, K_2_CO_3_, Dioxane:H_2_O (*v*/*v* = 4:1), 80 °C, 3 h, 80% yield.

Compound **10** was prepared according to Scheme 3. Commercially available 5-bromo-2-methylpyridin-3-amine (**36**) were sulfonylated to afford **37**. Conventional Suzuki coupling of **37** and 1-methylpyrazole-4-boronic acid pinacol ester (**30**) afforded compound **10**.

**Scheme 4:**
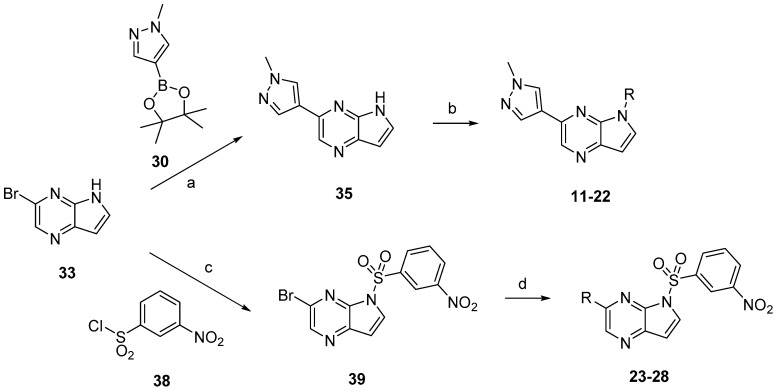
The synthesis route of compounds **11**–**28**. Reagents and conditions: (**a**) Pd(dppf)Cl_2_, K_2_CO_3_, Dioxane:H_2_O (*v*/*v* = 4:1), 80 °C, 3 h, 82% yield; (**b**) NaH, DMF, R-SO_2_Cl, r.t., 1–4 h, 72–87% yield; (**c**) NaH, DMF, r.t., 2 h, 80% yield; (**d**) Pd(dppf)Cl_2_, K_2_CO_3_, Dioxane:H_2_O (*v*/*v* = 4:1), 80 °C, 2–4 h, 81–92% yield.

Compounds **11**–**28** were prepared according to the procedures outlined in Scheme 4. Compound **33** was first treated via Suzuki coupling then sulfonylated with corresponding sulfonyl chlorides to afford compounds **11**–**22**. Correspondingly, compound **33** firstly was sulfonylated with 3-nitrobenzenesulfonyl chloride (**38**) then treated via Suzuki coupling with corresponding boric acid or boric acid ester to afford compounds **23**–**28**.

^1^H-NMR (400 MHz) and ^13^C-NMR (125 MHz or 151 MHz) spectra were recorded by using a Varian Mercury-400, a Mercury-500 and a Mercury-600 High Performance Digital FT-NMR spectrometer (Varian, Palo Alto, CA, USA) with tetramethylsilane (TMS) as an internal standard. Abbreviations for peak patterns in NMR spectra: br = broad, s = singlet, d = doublet, and m = multiplet. Low-resolution mass spectra were obtained with a Finnigan LCQ Deca XP mass spectrometer using a CAPCELL PAK C18 (50mm × 2.0 mm, 5 ZM) (Finnigan, Palo Alto, CA, USA) or an Agilent ZORBAX Eclipse XDB C18 (50 mm × 2.1 m, 5 ZM) (Agilent, Santa Clara, CA, USA) in positive or negative electrospray mode. Purity of all compounds was determined by analytical Gilson high-performance liquid chromatography (HPLC) using an YMC ODS3 column (50 mm × 4.6 mm, 5 ZM). Conditions were as follows: CH3CN/H2O eluent at 2.5 mL·min^−1^ flow (containing 0.1% trifluoroacetic acid (TFA)) at 35 °C, 8 min, gradient 5% CH3CN to 95% CH3CN, monitored by UV absorption at 214 nm and 254 nm. TLC analysis was carried out with glass precoated silica gel GF254 plates (Jiangyou, Qingdao, Shandong, China). TLC spots were visualized under UV light. Flash column chromatography was performed with a Teledyne ISCO CombiFlash Rf system (Teledyne, Huntsville, AL, USA). All solvents and reagents were used directly as obtained commercially unless otherwise noted. Anhydrous dimethylformamide was purchased from Acros (Shanghai, China) and was used without further drying. All air and moisture sensitive reactions were carried out under an atmosphere of dry Argon with heat-dried glassware and standard syringe techniques.

*6-(1-Methyl-1H-pyrazol-4-yl)-1H-pyrazolo[4,3-b]pyridine* (**31**). A solution of 6-bromo-1*H*-pyrazolo[4,3-*b*]pyridine (**29**) (330mg, 1.52 mmol), 1-methyl-4-(4,4,5,5-tetramethyl-1,3,2-dioxaborolan-2-yl)-1*H*-pyrazole (**30**) (378 mg, 1.82 mmol), PdCl_2_(dppf)-CH_2_Cl_2_ (61.9 mg, 80 µmol) and K_2_CO_3_ (628 mg, 4.54 mmol) in 1,4-dioxane:water (15 mL, 2:1, v) in a microwave tube was flushed with N_2_ for 5 min then sealed. The tube was heated at 80 °C for 2 h. Then the reaction mixture was evaporated to dryness. The residue was purified by flash chromatography to give **31** (260 mg, 86% yield); LC–MS *m*/*z* (ESI) found (M + H)^+^ 200.0 (M + H)^+^; ^1^H-NMR (400 MHz, DMSO-*d*_6_) δ13.29 (s, 1H), 8.80 (s, 1H), 8.36 (s, 1H), 8.24 (s, 1H), 8.07 (s, 2H), 3.90 (s, 3 H).

*6-(1-Methyl-1H-pyrazol-4-yl)-1-(phenylsulfonyl)-1H-pyrazolo[4,3-b]pyridine* (**4**). Sodium hydride (29 mg, 1.2 mmol) was suspended in 3 mL of anhydrous DMF. 6-(1-methyl-1*H*-pyrazol-4-yl)-1*H*-pyrazolo[4,3-*b*]pyridine (**31**) (160 mg, 0.8 mmol) was dissolved in 5 mL of anhydrous DMF was slowly added dropwise, and the mixture was stirred for 30 min benzenesulfonyl chloride (214 mg, 0.94 mmol) dissolved in 5 mL of anhydrous DMF was slowly added in drops, and the mixture was stirred for 4 h at room temperature. The reaction solution was poured to 0.1 N hydrochloric acid, which was then brought to basic using aqueous sodium bicarbonate solution, and extracted with ethyl acetate. The organic layer was collected, and distilled under reduced pressure. The remaining substance was purified by flash column chromatography to give **4** (224 mg, 82% yield); LC-MS *m*/*z* (ESI) found (M + H)^+^ 340.0 (M + H)^+^; ^1^H-NMR (400 MHz, CDCl_3_) δ 8.84 (d, *J* = 1.7 Hz, 1 H), 8.49 (s, 1 H), 8.38 (s, 1 H), 8.02 (d, *J* = 7.4 Hz, 2 H), 7.94 (s, 1H), 7.86 (s, 1H), 7.62 (t, *J* = 7.4 Hz, 1 H), 7.51 (t, *J* = 7.4 Hz, 2 H), 4.03 (s, 3 H). ^13^C-NMR (126 MHz, CDCl_3_) δ 146.75, 142.02, 141.60, 137.29, 137.20, 134.61, 134.41, 129.42 (C × 2), 128.85, 128.03, 127.72 (C × 2), 119.32, 116.03, 39.38. Retention time 2.95 min, >98% purity.

Compounds **5**–**7** were prepared with a similar procedure as that used for **4**.

*1-(Benzylsulfonyl)-6-(1-methyl-1H-pyrazol-4-yl)-1H-pyrazolo[4,3-b]pyridine* (**5**). LC–MS *m*/*z* (ESI) found (M + H)^+^ 354.1 (M + H)^+^; ^1^H-NMR (400 MHz, CDCl_3_) δ 8.72 (d, *J* = 1.9 Hz, 1H), 8.50 (d, *J* = 0.8 Hz, 1H), 7.74 (d, *J* = 0.8 Hz, 1H), 7.69 (dd, *J* = 1.9, 0.8 Hz, 1H), 7.67 (s, 1H), 7.11–7.03 (m, 3H), 6.97 (dd, *J* = 7.9, 1.6 Hz, 2H), 4.70 (s, 2H), 4.00 (s, 3H). ^13^C-NMR (126 MHz, CDCl_3_) δ 146.47, 141.71, 140.45, 137.16, 136.00, 130.55 (C × 2), 129.49, 128.56 (C × 2), 128.37, 127.73, 126.14, 119.12, 115.27, 60.31, 39.32. Retention time 2.97 min, >98% purity.

*1-Benzyl-6-(1-methyl-1H-pyrazol-4-yl)-1H-pyrazolo[4,3-b]pyridine* (**6**). LC–MS *m*/*z* (ESI) found (M + H)^+^ 290.1 (M + H)^+^; ^1^H-NMR (400 MHz, CDCl_3_) δ 8.73 (d, *J* = 1.8 Hz, 1H), 8.28 (d, *J* = 0.9 Hz, 1H), 7.81 (s, 1H), 7.72 (s, 1H), 7.64–7.59 (m, 1H), 7.38–7.30 (m, 3H), 7.25–7.21 (m, 2H), 5.64 (s, 2H), 4.00 (s, 3H). ^13^C-NMR (126 MHz, CDCl_3_) δ 140.67, 140.55, 137.55, 137.52, 137.10, 135.76, 131.18, 128.82 (C × 2), 128.54, 127.92, 127.73 (C × 2), 121.94, 101.51, 47.90, 39.22. Retention time 3.05 min, 98.25% purity.

*6-(1-Methyl-1H-pyrazol-4-yl)-1-phenethyl-1H-pyrazolo[4,3-b]pyridine* (**7**). LC–MS *m*/*z* (ESI) found (M + H)^+^ 304.1 (M + H)^+^; ^1^H-NMR (400 MHz, CDCl_3_) δ 8.74 (d, *J* = 1.9 Hz, 1H), 8.06 (d, *J* = 1.0 Hz, 1H), 7.96 (s, 1H), 7.88 (s, 1H), 7.75 (s, 1H), 7.32–7.24 (m, 3H), 7.14–7.10 (m, 2H), 4.68 (t, *J* = 7.3 Hz, 2H), 4.01 (s, 3H), 3.35 (t, *J* = 7.3 Hz, 2H). ^13^C-NMR (126 MHz, CDCl_3_) δ 153.72, 147.61, 145.87, 142.01, 140.34, 139.20, 137.77, 137.60, 136.48, 129.91, 129.02, 120.95, 110.86, 107.81, 105.68, 64.3, 39.36, 34.2. Retention time 3.08 min, >98% purity.

*6-(1-Methyl-1H-pyrazol-4-yl)-1H-pyrrolo[3,2-b]pyridine* (**34**). A solution of 6-bromo-1*H*-pyrrolo[3,2-*b*]pyridine (**32**) (100 mg, 0.51 mmol), 1-methyl-4-(4,4,5,5-tetramethyl-1,3,2-dioxaborolan-2-yl)-1*H*-pyrazole (**30**) (116 mg, 0.56 mmol), PdCl_2_(dppf)-CH_2_Cl_2_ (37 mg, 50 µmol) and K_2_CO_3_ (140 mg, 1.01 mmol) in 1,4-dioxane:water(6 mL, 2:1, v) in a microwave tube was flushed with N_2_ for 5 mins then sealed. The tube was heated at 80 °C for 3 h. Then the reaction mixture was evaporated to dryness. The residue was purified by flash chromatography to give **34** (90 mg, 89% yield); LC–MS *m*/*z* (ESI) found (M + H)^+^ 199.1 (M + H)^+^; ^1^H-NMR (400 MHz, DMSO-*d*_6_) δ 11.31 (brs, 1H), 8.59 (s, 1H), 8.21 (s, 1H), 7.93 (s, 1H), 7.87 (s, 1H), 7.60 (t, *J* = 2.8 Hz, 1H), 6.53 (t, *J* = 2.8 Hz, 1H), 3.88 (s, 3H).

*6-(1-Methyl-1H-pyrazol-4-yl)-1-(phenylsulfonyl)-1H-pyrrolo[3,2-b]pyridine* (**8**). Sodium hydride (7 mg, 0.28 mmol) was suspended in 3 mL of anhydrous DMF. 6-(1-methyl-1*H*-pyrazol-4-yl)-1*H*-pyrrolo[3,2-*b*]pyridine (**34**) (50 mg, 0.25 mmol) was dissolved in 3 mL of anhydrous DMF was slowly added dropwise, and the mixture was stirred for 30 min benzenesulfonyl chloride (38 μL, 0.28 mmol) dissolved in 3 mL of anhydrous DMF was slowly added in drops, and the mixture was stirred for 2 h at room temperature. The reaction solution was poured to 0.1 N hydrochloric acid, which was then brought to basic using aqueous sodium bicarbonate solution, and extracted with ethyl acetate. The organic layer was collected, and distilled under reduced pressure. The remaining substance was purified by flash column chromatography to give **8** (70 mg, 82% yield); LC–MS *m*/*z* (ESI) found (M + H)^+^ 339.1 (M + H)^+^; ^1^H-NMR (400 MHz, CDCl_3_) δ 8.70 (d, *J* = 1.7 Hz, 1H), 8.32 (d, *J* = 1.2 Hz, 1H), 7.91 (s, 1H), 7.89 (t, *J* = 1.7 Hz, 1H), 7.87–7.85 (m, 1H), 7.77 (d, *J* = 3.8 Hz, 2H), 7.63–7.56 (m, 1H), 7.49 (t, *J* = 7.7 Hz, 2H), 6.88 (dd, *J* = 3.8, 0.7 Hz, 1H), 4.02 (s, 3H). ^13^C-NMR (126 MHz, CDCl_3_) δ 147.08, 144.47, 138.00, 136.93, 134.28, 129.53 (C × 2), 129.25, 128.86, 127.36, 126.70 (C × 2), 124.81, 120.18, 117.08, 110.45, 39.25. Retention time 2.92 min, >98% purity.

Compounds **9** were prepared with a similar procedure as that used for **8**.

*3-(1-Methyl-1H-pyrazol-4-yl)-5-(phenylsulfonyl)-5H-pyrrolo[2,3-b]pyrazine* (**9**). LC–MS *m*/*z* (ESI) found (M + H)^+^ 340.0 (M + H)^+^; ^1^H-NMR (400 MHz, CDCl_3_) δ 8.71 (s, 1H), 8.28–8.19 (m, 2H), 8.09 (s, 1H), 8.03 (s, 1H), 7.94 (d, *J* = 4.1 Hz, 1H), 7.62 (d, *J* = 7.4 Hz, 1H), 7.54 (t, *J* = 7.7 Hz, 2H), 6.80 (d, *J* = 4.1 Hz, 1H), 4.04 (s, 3H). ^13^C-NMR (126 MHz, CDCl_3_) δ 142.22, 140.54, 139.05, 138.04, 137.96, 137.77, 134.47, 129.14 (C × 2), 129.10, 128.96, 128.24 (C × 2), 120.98, 106.58, 39.37. Retention time 2.99 min, >99% purity.

*N-(5-Bromo-2-methylpyridin-3-yl)benzenesulfonamide* (**37**). To a stirred solution of the 5-bromo-2-methylpyridin-3-amine (**36**) (200 mg, 1.07 mmol) in anhydrous dichloromethane (15 mL) was added benzenesulfonyl chloride (152 μL, 1.12 mmol). After 1 h, The mixture was then partially concentrated in vacuo, diluted with EtOAc (40 mL) and saturated NaHCO_3_ solution (20 mL) and partitioned. The aqueous layer was extracted with EtOAc (2 × 20 mL). The combined organic layers were dried (Na_2_SO_4_), filtered and concentrated to afford **37** (300 mg, 85% yield); LC–MS *m*/*z* (ESI) found (M + H)^+^ 328.1 (M + H)^+^; ^1^H-NMR (400 MHz, CDCl_3_) δ 8.37 (d, *J* = 2.1 Hz, 1H), 7.92 (d, *J* = 2.1 Hz, 1H), 7.82–7.76 (m, 2H), 7.67–7.61 (m, 1H), 7.56–7.49 (m, 2H), 2.17 (s, 3H).

*N-(2-Methyl-5-(1-methyl-1H-pyrazol-4-yl)pyridin-3-yl)benzenesulfonamide* (**10**). A solution of *N*-(5-bromo-2-methylpyridin-3-yl)benzenesulfonamide (**37**) (100 mg, 0.31 mmol), 1-methyl-4-(4,4,5,5-tetramethyl-1,3,2-dioxaborolan-2-yl)-1*H*-pyrazole (**30**) (70 mg, 0.34 mmol), PdCl_2_(dppf)-CH_2_Cl_2_ (20 mg, 24 µmol) and K_2_CO_3_ (84 mg, 0.61 mmol) in 1,4-dioxane:water(15 mL, 2:1, v) in a microwave tube was flushed with N_2_ for 5 mins then sealed. The tube was heated at 80 °C for 3 h. Then the reaction mixture was evaporated to dryness. The residue was purified by flash chromatography to give **10** (80 mg, 80% yield); LC–MS *m*/*z* (ESI) found (M + H)^+^ 329.1 (M + H)^+^; ^1^H-NMR (400 MHz, DMSO-*d*_6_) δ 8.45 (d, *J* = 2.0 Hz, 1H), 7.78–7.75 (m, 2H), 7.73 (d, *J* = 1.4 Hz, 1H), 7.71 (s, 1H), 7.67 (s, 1H), 7.59 (t, *J* = 7.5 Hz, 1H), 7.47 (t, *J* = 7.5 Hz, 2H), 5.30 (s, 1H), 3.97 (s, 3H), 2.16 (s, 3H). ^13^C-NMR (126 MHz, CDCl_3_) δ 149.37, 143.60, 139.24, 136.71, 133.43, 131.00, 129.32 (C × 2), 128.46, 127.44, 127.31, 127.03 (C × 2), 118.85, 39.23, 20.03. Retention time 2.54 min, >98% purity.

Compounds **11**–**28** were prepared with a similar procedure as that used for **8**.

*3-(1-Methyl-1H-pyrazol-4-yl)-5H-pyrrolo[2,3-b]pyrazine* (**35**). LC–MS *m*/*z* (ESI) found (M + H)^+^ 199.1 (M + H)^+^; ^1^H-NMR (400 MHz, DMSO-*d*_6_) δ 9.30 (s, 1H), 8.72 (s, 1H), 8.11 (s, 1H), 7.97 (s, 1H), 7.28 (s, 1H), 6.74 (dd, *J* = 3.7, 1.9 Hz, 1H), 4.02 (s, 3H).

*5-(Cyclopentylsulfonyl)-3-(1-methyl-1H-pyrazol-4-yl)-5H-pyrrolo[2,3-b]pyrazine* (**11**). LC–MS *m*/*z* (ESI) found (M + H)^+^ 311.2 (M + H)^+^; ^1^H-NMR (400 MHz, CDCl_3_) δ 8.80 (s, 1H), 8.09 (d, *J* = 0.8 Hz, 1H), 8.07 (d, *J* = 0.8 Hz, 1H), 7.83 (d, *J* = 4.1 Hz, 1H), 6.83 (d, *J* = 4.1 Hz, 1H), 4.03 (s, 3H), 3.16–3.01 (m, 1H), 2.20 (m, 2H), 2.01 (m, 2H), 1.96–1.62 (m, 4H). ^13^C-NMR (126 MHz, CDCl_3_) δ 142.25, 140.70, 139.08, 138.03, 137.65, 129.80, 129.30, 120.83, 105.69, 64.00, 40.57, 27.62 (C × 2), 25.86 (C × 2). Retention time 2.57 min, >99% purity.

*5-(Cyclohexylsulfonyl)-3-(1-methyl-1H-pyrazol-4-yl)-5H-pyrrolo[2,3-b]pyrazine* (**12**). LC–MS *m*/*z* (ESI) found (M + H)^+^ 345.1 (M + H)^+^; ^1^H-NMR (400 MHz, CDCl_3_) δ 8.81 (s, 1H), 8.07 (d, *J* = 0.7 Hz, 1H), 8.05 (d, *J* = 0.7 Hz, 1H), 7.84 (d, *J* = 4.0 Hz, 1H), 6.85 (d, *J* = 4.0 Hz, 1H), 4.01 (s, 3H), 3.82 (m, 1H), 2.01–1.85 (m, 4H), 1.69 (dd, *J* = 10.9, 4.8 Hz, 2H), 1.32–1.17 (m, 4H). Retention time 2.59 min, 98.54% purity.

*2-((3-(1-Methyl-1H-pyrazol-4-yl)-5H-pyrrolo[2,3-b]pyrazin-5-yl)sulfonyl)benzonitrile* (**13**). LC–MS *m*/*z* (ESI) found (M + H)^+^ 364.0 (M + H)^+^; ^1^H-NMR (400 MHz, CDCl_3_) δ 8.71 (s, 1H), 8.66–8.62 (m, 1H), 8.16 (d, *J* = 4.2 Hz, 1H), 8.01–7.96 (m, 2H), 7.89–7.83 (m, 2H), 7.77 (dd, *J* = 7.6, 1.3 Hz, 1H), 6.89 (d, *J* = 4.2 Hz, 1H), 4.02 (s, 3H). ^13^C-NMR (126 MHz, CDCl_3_) δ 142.29, 140.40, 139.90, 139.20, 138.37, 137.78, 135.52, 134.39, 132.82, 131.76, 130.15, 129.00, 120.65, 115.00, 111.37, 106.99, 39.36. Retention time 3.04 min, >99% purity.

*5-((4-Chlorophenyl)sulfonyl)-3-(1-methyl-1H-pyrazol-4-yl)-5H-pyrrolo[2,3-b]pyrazine* (**14**). HPLC 96.66%; LC–MS *m*/*z* (ESI) found (M + H)^+^ 373.0 (M + H)^+^; ^1^H-NMR (400 MHz, CDCl_3_) δ 8.73 (s, 1H), 8.19 (d, *J* = 8.5 Hz, 2H), 8.09 (s, 1H), 8.03 (s, 1H), 7.91 (d, *J* = 4.1 Hz, 1H), 7.51 (d, *J* = 8.5 Hz, 2H), 6.82 (d, *J* = 4.1 Hz, 1H), 4.05 (s, 3H). ^13^C-NMR (126 MHz, CDCl_3_) δ 142.31, 141.36, 140.46, 139.06, 138.17, 137.77, 136.36, 129.69 (C × 2), 129.53 (C × 2), 128.92, 128.89, 120.87, 106.91, 39.39. Retention time 2.98 min, 96.66% purity.

*5-((2,4-Difluorophenyl)sulfonyl)-3-(1-methyl-1H-pyrazol-4-yl)-5H-pyrrolo[2,3-b]pyrazine* (**15**). LC–MS *m*/*z* (ESI) found (M + H)^+^ 375.1 (M + H)^+^; ^1^H-NMR (400 MHz, CDCl_3_) δ 8.71 (s, 1H), 8.44 (td, *J* = 8.5, 6.0 Hz, 1H), 8.01 (dd, *J* = 4.1, 1.3 Hz, 1H), 7.99 (d, *J* = 0.7 Hz, 1H), 7.91 (s, 1H), 7.16–7.10 (m, 1H), 6.90 (ddd, *J* = 10.4, 8.5, 2.4 Hz, 1H), 6.85 (d, *J* = 4.1 Hz, 1H), 4.02 (s, 3H). ^13^C-NMR (126 MHz, CDCl_3_) δ 168.08, 166.01, 161.34, 159.25, 142.19, 140.38, 139.07, 138.16, 137.76, 133.92, 129.52, 128.76, 120.74, 112.10, 106.53, 39.34. Retention time 2.97 min, 94.53% purity.

*5-((3,4-Dichlorophenyl)sulfonyl)-3-(1-methyl-1H-pyrazol-4-yl)-5H-pyrrolo[2,3-b]pyrazine* (**16**). LC–MS *m*/*z* (ESI) found (M + H)^+^ 407.0 (M + H)^+^; ^1^H-NMR (400 MHz, CDCl_3_) δ 8.75 (s, 1H), 8.50 (d, *J* = 2.2 Hz, 1H), 8.10 (d, *J* = 0.7 Hz, 1H), 8.06 (s, 1H), 8.03 (dd, *J* = 8.5, 2.2 Hz, 1H), 7.90 (d, *J* = 4.1 Hz, 1H), 7.62 (d, *J* = 8.5 Hz, 1H), 6.84 (d, *J* = 4.1 Hz, 1H), 4.06 (s, 3H). ^13^C-NMR (126 MHz, CDCl_3_) δ 142.44, 140.33, 139.75, 139.07, 138.37, 137.69, 137.34, 133.79, 131.27, 130.76, 129.06, 128.62, 127.09, 120.71, 107.13, 39.38. Retention time 3.02 min, >99% purity.

*3-(1-Methyl-1H-pyrazol-4-yl)-5-((3-nitrophenyl)sulfonyl)-5H-pyrrolo[2,3-b]pyrazine* (**17**). LC–MS *m*/*z* (ESI) found (M + H)^+^ 384.2 (M + H)^+^; ^1^H-NMR (400 MHz, CDCl_3_) δ 9.40 (t, *J* = 2.1 Hz, 1H), 8.77 (s, 1H), 8.49 (dd, *J* = 10.8, 8.0 Hz, 2H), 8.24 (s, 1H), 8.06 (s, 1H), 7.92 (d, *J* = 4.2 Hz, 1H), 7.78 (t, *J* = 8.0 Hz, 1H), 6.86 (dd, *J* = 4.2, 0.7 Hz, 1H), 4.08 (s, 3H). ^13^C-NMR (126 MHz, CDCl_3_) δ 148.09, 142.68, 140.32, 139.87, 139.00, 138.45, 137.37, 133.39, 130.67, 129.68, 128.93, 128.39, 124.61, 120.43, 107.41, 39.37. Retention time 3.01 min, >99% purity.

*3-(1-Methyl-1H-pyrazol-4-yl)-5-((4-nitrophenyl)sulfonyl)-5H-pyrrolo[2,3-b]pyrazine* (**18**). LC–MS *m*/*z* (ESI) found (M + H)^+^ 384.0 (M + H)^+^; ^1^H-NMR (400 MHz, CDCl_3_) δ 8.75 (s, 1H), 8.48–8.44 (m, 2H), 8.37 (d, *J* = 8.8 Hz, 2H), 8.10 (s, 1H), 8.03 (s, 1H), 7.91 (d, *J* = 4.1 Hz, 1H), 6.87 (d, *J* = 4.1 Hz, 1H), 4.06 (s, 3H). Retention time 3.02 min, 98.86% purity.

*3-((3-(1-Methyl-1H-pyrazol-4-yl)-5H-pyrrolo[2,3-b]pyrazin-5-yl)sulfonyl)aniline* (**19**). LC–MS *m*/*z* (ESI) found (M + H)^+^ 354.1 (M + H)^+^; ^1^H-NMR (600 MHz, CDCl_3_) δ 9.05 (d, *J* = 1.9 Hz, 1H), 8.67 (s, 1H), 8.17–8.14 (m, 2H), 8.06 (dd, *J* = 8.4, 0.8 Hz, 1H), 8.04 (d, *J* = 0.8 Hz, 1H), 7.98 (d, *J* = 4.1 Hz, 1H), 7.42 (d, *J* = 8.4 Hz, 1H), 6.80 (d, *J* = 4.1 Hz, 1H), 4.05 (s, 3H). Retention time 2.79 min, 94.56% purity.

*4-((3-(1-Methyl-1H-pyrazol-4-yl)-5H-pyrrolo[2,3-b]pyrazin-5-yl)sulfonyl)aniline* (**20**). LC–MS *m*/*z* (ESI) found (M + H)^+^ 355.07 (M + H)^+^; ^1^H-NMR (400 MHz, CDCl_3_) δ 8.69 (s, 1H), 8.09 (d, *J* = 0.8 Hz, 1H), 8.03 (s, 1H), 8.01 (d, *J* = 2.1 Hz, 1H), 7.99 (d, *J* = 2.1 Hz, 1H), 7.93 (d, *J* = 4.1 Hz, 1H), 6.76 (d, *J* = 4.1 Hz, 1H), 6.66 (d, *J* = 2.0 Hz, 1H), 6.64 (d, *J* = 2.0 Hz, 1H), 4.04 (s, 3H). Retention time 2.75 min, 98.68% purity.

*N-(3-((3-(1-Methyl-1H-pyrazol-4-yl)-5H-pyrrolo[2,3-b]pyrazin-5-yl)sulfonyl)phenyl)acetamide* (**21**). HPLC 94.55%; LC–MS *m*/*z* (ESI) found (M + H)^+^ 396.1 (M + H)^+^; ^1^H-NMR (400 MHz, CDCl_3_) δ 8.72 (s, 1H), 8.61 (s, 1H), 8.20 (s, 1H), 8.06 (s, 1H), 7.91 (t, *J* = 6.0, 6.3 Hz, 2H), 7.66 (d, *J* = 8.2 Hz, 1H), 7.47 (d, *J* = 8.2 Hz, 1H), 7.44 (d, *J* = 12.3 Hz, 1H), 6.80 (d, *J* = 4.1 Hz, 1H), 4.01 (s, 3H), 2.04 (s, 3H). Retention time 3.12 min, 94.55% purity.

*N-(3-((3-(1-Methyl-1H-pyrazol-4-yl)-5H-pyrrolo[2,3-b]pyrazin-5-yl)sulfonyl)phenyl)propionamide* (**22**). LC–MS *m*/*z* (ESI) found (M + H)^+^ 410.1 (M + H)^+^; ^1^H-NMR (400 MHz, CDCl_3_) δ 8.71 (s, 1H), 8.61 (s, 1H), 8.22 (s, 1H), 8.07 (s, 1H), 7.92 (t, *J* = 6.1 Hz, 2H), 7.67 (d, *J* = 8.1 Hz, 1H), 7.47 (d, *J* = 8.1 Hz, 1H), 7.42 (d, *J* = 12.5 Hz, 1H), 6.81 (d, *J* = 4.1 Hz, 1H), 4.04 (s, 3H), 2.41 (q, *J* = 7.6 Hz, 2H), 1.24 (t, *J* = 7.6 Hz, 3H). Retention time 3.13 min, 97.72% purity.

*1-(4-(5-((3-Nitrophenyl)sulfonyl)-5H-pyrrolo[2,3-b]pyrazin-3-yl)-1H-pyrazol-1-yl)ethan-1-one* (**23**). LC–MS *m*/*z* (ESI) found (M + H)^+^ 412.0 (M + H)^+^; ^1^H-NMR (400 MHz, CDCl_3_) δ 9.28 (s, 1H), 8.91 (s, 1H), 8.86 (s, 1H), 8.58 (d, *J* = 8.2 Hz, 1H), 8.51 (d, *J* = 8.2 Hz, 1H), 8.40 (s, 1H), 8.02 (d, *J* = 4.2 Hz, 1H), 7.81 (t, *J* = 8.2 Hz, 1H), 6.91 (d, *J* = 4.2 Hz, 1H), 2.83 (s, 3H). ^13^C-NMR (126 MHz, CDCl_3_) δ 169.38, 148.24, 142.03, 140.64, 140.34, 140.19, 139.73, 139.00, 133.57, 130.68, 129.81, 129.04, 126.08, 124.04, 123.66, 107.39, 21.67. Retention time 2.64 min, 90.95% purity.

*1-(4-(5-((3-Nitrophenyl)sulfonyl)-5H-pyrrolo[2,3-b]pyrazin-3-yl)-1H-pyrazol-1-yl)propan-1-one* (**24**). LC–MS *m*/*z* (ESI) found (M + H)^+^ 426.3 (M + H)^+^; ^1^H-NMR (400 MHz, CDCl_3_) δ 9.27 (t, *J* = 2.0, 2.0 Hz, 1H), 8.91 (d, *J* = 0.8 Hz, 1H), 8.86 (s, 1H), 8.61–8.56 (m, 1H), 8.53–8.48 (m, 2H), 8.38 (d, *J* = 0.8 Hz, 1H), 8.32 (s, 1H), 8.02 (d, *J* = 4.2 Hz, 1H), 6.91 (d, *J* = 4.2 Hz, 1H), 3.27 (q, *J* = 7.4 Hz, 2H), 1.38 (t, *J* = 7.4 Hz, 3H). ^13^C-NMR (126 MHz, CDCl_3_) δ 172.88, 148.25, 141.86, 140.77, 140.28, 139.75, 139.00, 138.69, 133.60, 130.66, 129.74, 129.04, 126.18, 124.36, 124.02, 123.35, 107.39, 29.70. Retention time 2.65 min, 95.32% purity.

*1-(4-(5-((3-Nitrophenyl)sulfonyl)-5H-pyrrolo[2,3-b]pyrazin-3-yl)-1H-pyrazol-1-yl)propan-2-one* (**25**). LC–MS *m*/*z* (ESI) found (M + H)^+^ 426.2 (M + H)^+^; ^1^H-NMR (400 MHz, CDCl_3_) δ 9.40 (t, *J* = 2.0 Hz, 1H), 8.79 (s, 1H), 8.48 (m, 2H), 8.30 (d, *J* = 0.8 Hz, 1H), 8.14 (d, *J* = 0.8 Hz, 1H), 7.95 (d, *J* = 4.1 Hz, 1H), 7.79 (t, *J* = 8.0 Hz, 1H), 6.87 (d, *J* = 4.1 Hz, 1H), 5.11 (s, 2H), 2.29 (s, 3H). ^13^C-NMR (151 MHz, CDCl_3_) δ 201.10, 147.99, 142.15, 140.19, 139.74, 139.23, 138.40, 138.03, 133.42, 130.72, 130.44, 128.98, 128.59, 124.75, 121.22, 107.31, 61.19, 27.02. Retention time 2.66 min, 94.67% purity.

*1-(4-(5-((3-Nitrophenyl)sulfonyl)-5H-pyrrolo[2,3-b]pyrazin-3-yl)phenyl)ethan-1-one* (**26**). LC–MS *m*/*z* (ESI) found (M + H)^+^ 422.3 (M + H)^+^; ^1^H-NMR (400 MHz, CDCl_3_) δ 9.32 (t, *J* = 2.0 Hz, 1H), 9.12 (s, 1H), 8.60–8.55 (m, 1H), 8.53–8.48 (m, 1H), 8.25 (d, *J* = 8.4 Hz, 2H), 8.18 (d, *J* = 8.4 Hz, 2H), 8.09 (d, *J* = 4.2 Hz, 1H), 7.79 (t, *J* = 8.1 Hz, 1H), 6.96 (d, *J* = 4.2 Hz, 1H), 3.73 (d, *J* = 0.8 Hz, 3H). Retention time 2.65 min, 91.84% purity.

*(4-(5-((3-Nitrophenyl)sulfonyl)-5H-pyrrolo[2,3-b]pyrazin-3-yl)phenyl)methanol* (**27**). LC–MS *m*/*z* (ESI) found (M + H)^+^ 410.0 (M + H)^+^; ^1^H-NMR (400 MHz, CDCl_3_) δ 9.30 (t, *J* = 2.0 Hz, 1H), 9.02 (s, 1H), 8.57 (dt, *J* = 7.9, 1.5 Hz, 1H), 8.48 (ddd, *J* = 8.2, 2.0, 1.1 Hz, 1H), 8.13 (d, *J* = 1.5 Hz, 1H), 8.11 (s, 1H), 8.02 (d, *J* = 4.2 Hz, 1H), 7.77 (t, *J* = 8.1 Hz, 1H), 7.57 (d, *J* = 8.1 Hz, 2H), 6.91 (d, *J* = 4.2 Hz, 1H), 4.83 (s, 2H). ^13^C-NMR (126 MHz, CDCl_3_) δ 148.17, 147.07, 142.88, 140.39, 140.11, 139.84, 139.22, 135.31, 133.66, 130.68, 129.64, 128.97, 127.64 (C × 2), 127.20 (C × 2), 124.29, 107.25, 64.85. Retention time 2.71 min, 94.62% purity.

*3-(7-Methoxy-1H-indol-2-yl)-5-((3-nitrophenyl)sulfonyl)-5H-pyrrolo[2,3-b]pyrazine* (**28**). LC–MS *m*/*z* (ESI) found (M + H)^+^ 449.1 (M + H)^+^; ^1^H-NMR (400 MHz, CDCl_3_) δ 9.43 (s, 1H), 9.19 (s, 1H), 9.07 (s, 1H), 8.58 (s, 1H), 8.50 (s, 1H), 7.99 (s, 1H), 7.81 (s, 1H), 7.23–7.03 (m, 2H), 6.97–6.87 (m, 1H), 6.78 (d, *J* = 8.4 Hz, 1H), 4.12 (s, 3H). Retention time 2.94 min, 96.21% purity.

## 4. Conclusions

Starting with the scaffold of 1-sulfonylpyrazolo[4,3-*b*]pyridine discovered in c-Met inhibitor development, we conducted a hit optimization towards the inhibitory activity of FGFR1. We explored the SAR of this new chemotype on three parts, namely the scaffold, π–π stacking part and methyl pyrazolepart. We found that the scaffold is essential to the binding activity, and substituting the pyrazolo[4,3-*b*]pyridine with pyrrolo[2,3-*b*]pyrazine increases about of the inhibitory activity on FGFR1 about 10-fold. The π–π stacking part is also vital to the biological activity, showing greater importance of the steric effect than the electronic effect. In summary, we elaborated a series of FGFR inhibitors containing the novel scaffold of pyrrolo[2,3-*b*]pyrazine. This scaffold, together with the interesting shape of this phenotype, could help others to further develop selective FGFR inhibitors for cancer treatment.

## Figures and Tables

**Table 1:** Structures and FGFR1 enzymatic activities of compounds **4**–**7** ^a^.

Compound No.	Structure	FGFR1 Inhibition (%) ^b^
		10 μM
**4**	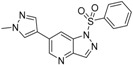	87.8
**5**	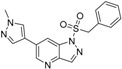	46.1
**6**	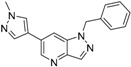	53
**7**	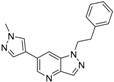	55.9

^a^ In our enzymatic assay, the IC_50_ value of AZD4547 on FGFR1 is 1.8 ± 0.1 nM (mean ± standard deviation). ^b^ Inhibition values are given as the mean (%) from two separate experiments. FGFR1: fibroblast growth factor receptor 1.

**Table 2:** Structures and FGFR1 enzymatic activities of compounds **8**–**10**.

Compound No.	Structure	FGFR1 Inhibition (%)	
		10 μM	1 μM
**4**	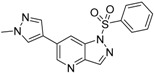	87.8	47.1
**8**	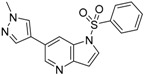	80.4	31.6
**9**	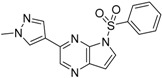	93.3	92.5
**10**	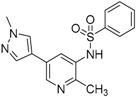	50.0	15.4

**Table 3:**
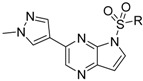
Structures and FGFR1 enzymatic activities of compounds **11**–**22**.

Compound No.	R	FGFR1 Inhibition (%)		
		10 μM	1 μM	0.1 μM
**11**	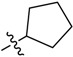	34.7	20.8	26.8
**12**	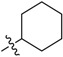	53.3	24.1	29.4
**9**	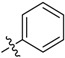	90.5	63.4	46
**13**	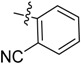	59.7	32.6	40.3
**14**	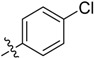	45.2	27.2	22
**15**	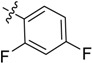	55.6	31.2	23.6
**16**	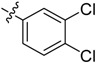	74.5	34.8	13.6
**17**	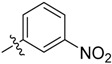	92.1	76.4	61.1
**18**	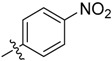	15.5	12.8	17.4
**19**	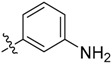	82.2	57.5	34.7
**20**	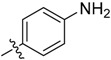	42.4	24.2	31.8
**21**	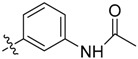	79	50.9	44.7
**22**	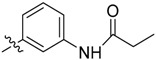	38.7	17.3	27.7

**Table 4:**
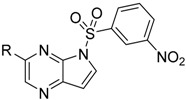
Structures and FGFR1 enzymatic activities of compounds **23**–**28**.

Compound No.	R	FGFR1 Inhibition (%)		
		1 μM	0.1 μM	0.01 μM
**17**	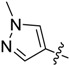	76.4	61.1	ND
**23**	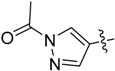	58.8	37.8	36.5
**24**	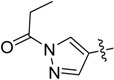	60.9	30.2	29.3
**25**	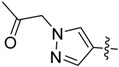	63.4	55.7	32.8
**26**	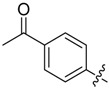	57.9	35.7	ND
**27**	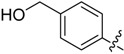	75.5	52.2	ND
**28**	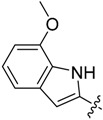	62.4	34	27.2

Note: ND = not determined.

**Table 5:** FGFR1 and c-Met enzymatic activities of compounds **17**, **25** and **27**.

Compound No.	FGFR1 IC_50_ (nM) ^a^	c-Met Inhibition (%)		
		1 μM	0.1 μM	0.01 μM
**17**	85.9 ± 21.2	30.6	−1.5	−5.4
**25**	45.9 ± 14.5	14.1	5.0	5.5
**27**	113.8 ± 5.8	30.1	8.4	1.1

^a^ IC_50_ values are given as the mean ± SD (nM) from two separate experiments. c-Met: hepatocyte growth factor receptor.
